# A complex pattern of chemokine receptor expression is seen in osteosarcoma

**DOI:** 10.1186/1471-2407-8-23

**Published:** 2008-01-24

**Authors:** Irene von Luettichau, Stephan Segerer, Alexandra Wechselberger, Mike Notohamiprodjo, Michaela Nathrath, Markus Kremer, Anna Henger, Roghieh Djafarzadeh, Stefan Burdach, Ralf Huss, Peter J Nelson

**Affiliations:** 1 Children's Hospital Medical Center, University of Technology, Munich, Germany; 2 Medical Policlinic, Ludwig-Maximilians University, Munich, Germany; 3 Institute of Pathology, Ludwig-Maximilians University, Munich, Germany; 4 Institute of Pathology GSF, Munich, Germany; 5 Division of Nephrology, Department of Internal Medicine, University of Michigan, Ann Arbor, MI, USA; 6 Clinical Cooperation Group "Osteosarcoma", GSF-National Research Centre for Environment and Health, Munich, Germany

## Abstract

**Background:**

Osteosarcoma is the most frequent bone tumor in childhood and adolescence. Patients with primary metastatic disease have a poor prognosis. It is therefore important to better characterize the biology of this tumor to define new prognostic markers or therapeutic targets for tailored therapy. Chemokines and their receptors have been shown to be involved in the development and progression of malignant tumors. They are thought to be active participants in the biology of osteosarcoma. The function of specific chemokines and their receptors is strongly associated with the biological context and microenvironment of their expression. In this report we characterized the expression of a series of chemokine receptors in the complex environment that defines osteosarcoma.

**Methods:**

The overall level of chemokine receptor mRNA expression was determined using TaqMan RT-PCR of microdissected archival patient biopsy samples. Expression was then verified at the protein level by immunohistochemistry using a series of receptor specific antibody reagents to elucidate the cellular association of expression.

**Results:**

Expression at the RNA level was found for most of the tested receptors. CCR1 expression was found on infiltrating mononuclear and polynuclear giant cells in the tumor. Cells associated with the lining of intratumoral vessels were shown to express CCR4. Infiltrating mononuclear cells and tumor cells both showed expression of the receptor CCR5, while CCR7 was predominantly expressed by the mononuclear infiltrate. CCR10 was only very rarely detected in few scattered infiltrating cells.

**Conclusion:**

Our data elucidate for the first time the cellular context of chemokine receptor expression in osteosarcoma. This is an important issue for better understanding potential chemokine/chemokine receptor function in the complex biologic processes that underlie the development and progression of osteosarcoma. Our data support the suggested involvement of chemokines and their receptors in diverse aspects of the biology of osteosarcoma, but also contradict aspects of previous reports describing the expression of these receptors in this tumor.

## Background

Osteosarcoma is a primary tumor of the bone that accounts for 5% of childhood cancers and represents the fifth most frequent tumor in young adults [1]. In 15–20% of cases metastasis are present at the time of diagnosis. An additional 20–25% of the patients develop metastasis during the course of disease and have a very poor prognosis despite achievements in multimodal therapy [1]. Currently osteosarcoma is classified according to histological criteria with osteoblastic, chondroblastic and fibroblastic being the most frequent predominant histological elements [2]. The prognosis for patients is predicted by evaluating their response to preoperative chemotherapy according to the criteria of Salzer-Kuntschik [3]. Additional markers of tumor characteristics would aid in classification of the tumor and potentially novel prognostic markers could be identified to stratify therapy according to the individual risk.

Chemokines are proinflammatory cytokines that are produced locally in tissues and function as the directional cues to sort, direct, and fine tune cell trafficking [4]. The receptors for chemokines are expressed on a variety of cells including tumor cells. Their expression on diverse types of cells associated with tumor progression and the omnipresence of their ligands has moved them into the focus of cancer research. Diverse biological roles for chemokines and their receptors in tumor growth and metastases have been identified [5-9]. These actions include: modulation of tumor angiogenesis, tumor sensitivity to apoptosis, tumor proliferation, control of matrix degradation and the directed invasion of malignant cells during tumor metastasis [5-9]. Chemokine biology is also central to the immunologic anti-tumor response through the recruitment of effector lymphocytes and the subsequent regulation of their effector function within tumor environments [10]. Data from breast carcinoma studies have suggested that the specific effects mediated through chemokines could be significantly different depending on the source of ligand or receptor expression [11]. In a recent retrospective osteosarcoma patient study, the mRNA expression of the receptors CXCR4, CCR7 and CCR10 by osteosarcoma tissue was linked to clinical outcome [12]. However, the expression and distribution of some receptors in osteosarcoma is still controversial [13]. Since osteosarcoma consists not only of the tumor cells derived from a mesenchymal origin, but also of numbers of infiltrating mononuclear cells [14] we attempted to define the source of the chemokine receptor expression in patient samples. Our results show diverse chemokine receptor expression by different cell types within the tumor environment.

## Methods

### Cell lines

#### Isolation of primary mesenchymal stem cells and the generation of clonal immortalized human progenitor cell lines

Primary human CD34^- ^stem cells were isolated from bone marrow of healthy donors (discarded material from pilot vials used after local consent) as previously described [15]. The immune phenotype of the primary and immortalized cells was monitored via FACS analysis throughout the course of experiments as previously described [15].

#### Osteoblast cell line

The human fetal osteoblast cell line FOB was purchased from American Type Culture Collection (Manassas, Virginia, USA). Cells were grown in Ham's F12/Dulbeccos modified Eagle (Gibco, Karsruhe, Germany) with 2.5 mM Glutamin, 0.3 mg/ml G418, and 10% FCS at the permissive temperature of 33°C and harvested at 80% confluency. To induce differentiation the cells were kept at the restrictive temperature of 39°C for 5–8 days. Cells were then harvested when no further proliferation could be observed and analyzed for expression of osteocalcin and osteopontin using real time RT-PCR Expression levels of these two genes in undifferentiated osteoblasts were compared with that seen following differentiation (data not shown). RNA was extracted from all cell lines using the RNeasy RNA extraction kit (Qiagen, Hilden, Germany) according to manufacturer's recommendations including DNAse digestion and followed by transcription (see below).

#### Patient samples

##### Osteosarcoma biopsies

Biopsies were taken from patients with osteosarcoma after informed consent of the local therapy center and with the approval of the local ethics committee (ethics committee of the medical faculty of the Technical University of Munich) and processed for routine diagnostic procedures. Discarded material was used for the research purposes that were carried out in compliance with the Helsinki Declaration. The samples were formaldehyde fixed, paraffin embedded and archived at room temperature. All sections of paraffin embedded biopsy tissue were subjected to microscopic directed manual micro dissection using morphological criteria prior to RNA extraction. A total of 27 samples were analyzed from 27 patients. Morphological diagnostics revealed osteosarcoma in all cases. Histological subtype was determined according to the WHO criteria [16]. The samples included: 15 osteoblastic sarcoma, one fibroblastic, four small cell, three chondroblastic, two teleangetatic, one round cell, and one mixed cell sarcomas. Two independent expert pathologists confirmed the histological diagnosis. RNA was additionally extracted from one osteoblastoma biopsy as a reference tissue. All osterosarcoma patients were treated according to the standardized treatment protocol of the German-Austrian-Swiss OS study group (COSS) active at the time of enrolment (COSS 86, COSS 91 and COSS 96 respectively). Response to chemotherapy was defined according to the criteria of Salzer-Kuntschik after tumor resection [3].

##### Normal bone tissue

Analysis of mature cortical bone was performed on anonymous archival material from the iliac crest obtained through the Institute of Pathology (LMU).

##### Tonsil tissue

Analysis of tonsil sections was used to demonstrate positive controls. Anonymous archival material from inflamed tonsils was obtained through the Institute of Pathology (LMU).

### RNA extraction method for paraffin embedded osteosarcoma tissue

Microdissected specimens from fixed osteosarcoma, bone tissue sections and tonsil tissue as well as sections from the osteoblastoma were deparafinized, rehydrated and harvested in 200 μl lysis buffer containing 2% Sodium dodecyl sulphate (SDS), 10 mmol/l Tris-HCL (ph 8,0), 0,1 mmol/l ethylenediaminetetraacetic acid (EDTA; ph 8,0), and 500 μg/l proteinase K (Sigma, Steinheim, Germany). Samples were incubated at 60°C for 16 hours. RNA was extracted using phenol/chloroform extraction. The pelleted RNA was resuspended in 10/20/30 μl of RNAse free H_2_O respectively depending on the amount of tissue available before RNA extraction.

Because chemokine receptor genes generally contain only one intron it is often difficult to generate good cDNA specific real time TaqMan PCR probes (requiring efficient amplicons of less than 100 nt). To increase flexibility in probe design, all RNA samples were routinely treated with DNase I to reduce contamination of genomic DNA prior to PCR analysis. Twenty-seven Kunitz units of RNAse free DNAse and 1/10 volume buffer RDD (Qiagen, Hilden, Germany) were added to each sample. After incubation for 15 minutes at room temperature, the DNAse was inactivated for 10 minutes at 65°C in presence of 2 mM EDTA.

The entire sample was then reverse transcribed in 20/30/40 μl volume depending on the amount of RNA, with first strand buffer, 5 mM DTT (Invitrogen, Scotland), 0,5 mM dNTP (Amersham Pharmacia, Freiburg, Germany), RNase inhibitor (RNasin, Promega, Mannheim, Germany) and 0.19 μg/ml Acrylamid (Ambion, Austin TX, USA), 21,5 μg/ml random hexamers (Roche, Mannheim, Germany) and 172 U reverse transcriptase (Superscript, Invitrogen) for 1 hour at 42°C.

### Real time RT-PCR for quantitative analysis of mRNA expression of chemokine receptors

Real time RT-PCR was performed on a TaqMan ABI 7000 Sequence Detection System^® ^(Applied Biosystems, Weiterstadt, Germany) using heat activated TaqDNA polymerase (Amplitaq Gold, Applied Biosystems) essentially as described [17]. After an initial hold of 2 min at 50°C and 10 min at 95°C the samples were cycled 40 times at 95°C for 15 sec and 60°C for 60 sec. Quantitative analysis of the cDNA was done following the ΔCt technique (expression level = 2^CT rRNA – CT gene of interest^). CT values indicate the first cycle with a detectable fluorescence signal in TaqMan RT-RCR. Gene expression that was negative after 40 cycles in repeated analysis was determined "not detectable". Very low positive signals (CT 38–40) were determined as "borderline" values indicating the procedural deviation in this range of expression where false positive results may occur and no clear analysis is possible. 18 S rRNA was used for normalization of cDNA content and amplified in parallel with the genes of interest. Sample gene expression was then normalized against rRNA expression levels. No template controls (NTC) were negative in all runs. All measurements were performed in duplicates. Commercially available pre-developed TaqMan reagents were used for hCCR1, hCCR2, hCCR4, hCCR5, hGAPDH and rRNA (PDAR). In addition, probes for the following receptors were generated as described elsewhere: hCCR7, hCXCR4, hCXCR3, hCXCR5, hCCR10 and hCX3CR1 [15,18,19]. All TaqMan reagents were obtained from Applied Biosystems, Warrington, UK.

### Immunohistochemistry

The tissue distribution of chemokine receptors CCR1, CCR4, CCR7, CXCR3, CXCR5, CCR5, and CCR10 was determined by immunohistochemistry on consecutive sections from formalin-fixed paraffin-embedded tissue. The use of antibodies directed against human CCR5 (MC5, a gift from M. Mack) and CXCR3 (Clone 1C6, BD Biosciences Pharmingen) were previously described on fixed tissues [19,20]. Antibodies against CCR10 and CCR4 were a gift from Millennium Pharmaceuticals Inc., (Cambridge, Massachusetts, USA) [15,18,19]. CCR1-specific antibody reagent was obtained commercially (Santa Cruz Biotechnology, Santa Cruz, California, USA, sc-6125) and used as described (Segerer et al. submitted manuscript). CXCR5-specific antibody was a gift from Elisabeth Kremmer (GSF, Munich, Germany). Sections of inflamed human tonsil were used as positive control material for the expression of CCR1, CCR5 and CCR7 [20]. Negative controls were isotype matched IgGs or goat non-immune serum in case of CCR4 and primary antibody was replaced by diluent.

The general protocol used for antigen retrieval from archival samples was performed as previously described [19]. In brief, slides were deparafinized in xylene and rehydrated in a series of graded ethanol. Slides were treated with 3% H2O2 to block endogenous peroxide. Antigen retrieval was performed by autoclaving in Antigen retrieval solution (Vector Laboratories, Burlingame, CA). Endogenous biotin was blocked by the Biotin/Avidin blocking kit (Vector). Primary antibodies were applied for 1 hour, followed by secondary biotinylated antibodies (Vector) and the ABC reagent (Vector). 3'3' Diaminobenzidine (DAB, Sigma, Taufkirchen, Germany) with metal enhancement (resulting in a black colour product) served as detection system. Slides were counterstained with methyl green, dehydrated and mounted. Analysis was performed using Leica DM6000 B microscope with the Leica software Application Suite AF 6000. A pathologist (R. H.) analyzed Chemokine receptor expression.

## Results

### Chemokine receptor mRNA expression in osteosarcoma biopsy samples

Following tumor surgery samples are generally fixed in formalin for diagnostic procedures. The study of gene expression in fresh tumor samples is often limited by the availability of snap frozen patient material. In order to make use of archived tumor material, a method was developed that allowed efficient extraction of RNA from archival formalin fixed osteosarcoma tumor samples following manual microdissection of the individual sections (see Material and Methods and [17]). In these studies TaqMan RT-PCR analysis was found to be ideally suited for the mRNA analysis, as it requires relatively small amplicons thus minimizing the effect of any fragmentation of mRNA that may have occurred during its extraction from archival samples.

Twenty-seven paraffin embedded archived specimens of childhood and adult osteosarcoma from diagnostic biopsies were analyzed for expression of chemokine receptor mRNA. Patient characteristics are summarized in Table 1. All samples were obtained before therapy, fixed and paraffin embedded. Unfortunately, not enough mRNA could be recovered from each sample to analyze all receptors in parallel. A subgroup of specific chemokine receptors were selected for detailed study based on mRNA and protein expression profiles from a series of osteosarcoma cell lines (data not shown) as well as from literature suggesting the involvement of specific receptors in cancer, cancer progression and osteosarcoma [8,12,21,22]. The mRNA expression levels of CCR1 (n = 21), CCR2 (n = 13), CCR4 (n = 22), CCR5 (n = 12), CCR7 (n = 23), CCR10 (n = 12), CXCR3 (n = 8), CXCR4 (n = 25), CXCR5 (n = 11) and CX3CR1 (n = 22) were evaluated using quantitative RT-PCR (see Materials and Methods).

**Table 1:** Patient Characteristics

**Patient number**		27
**Age at diagnosis**	<12	5
	13–20	12
	21–30	6
	31–40	1
	41-	3
**Sex**	male	8
	female	19
**Primary tumor site**	femur	18
	humerus	2
	others	7
**Outcome**	DOD	10
	CR1	14
	CR2	3
**Histology**	osteoblastic	15
	small cell	4
	chondroblastic	3
	teleangetatic	2
	round cell	1
	fibroblastic	1
	mixed	1
**Response to chemotherapy**	good	13
	poor	12
	unavailable	1*
**Relapse**	yes	11
	no	16
**Primary metastasis**	no	21
	lung	5
	other	1
**Survival time**	<1 year	4
	<4 years	4
	>4 years	19

* Tumour was removed before onset of chemotherapy

**Figure 1:**
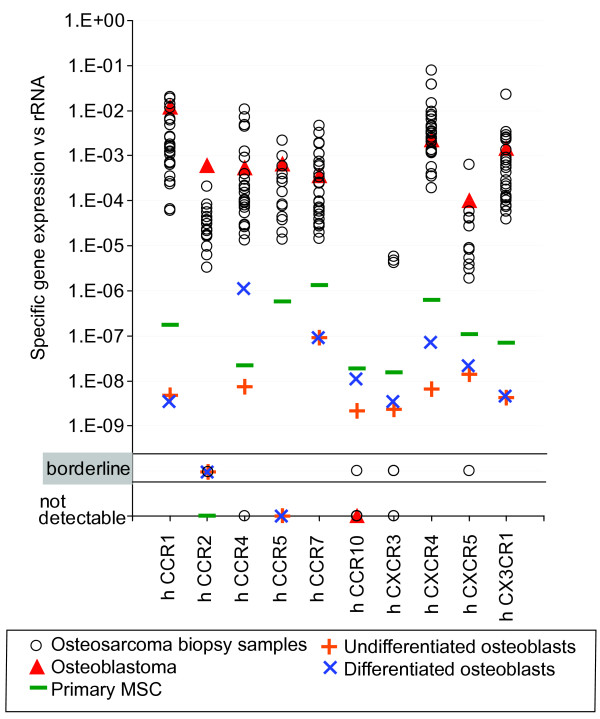
**mRNA expression analysis of chemokine receptors in osteosarcoma tumor biopsies and bone precursors**. The **open black circles **indicate values of chemokine receptor expression of mRNA isolates from osteosarcoma biopsy samples with each circle representing the value of a single sample. **Red Triangles **indicate the mRNA expression of the osteoblastoma. Median values of at least three independent RNA preparations are represented by **green hyphens **for primary MSC, undifferentiated (**orange cross**) and differentiated osteoblasts (**blue X**). The grey field indicates borderline detectable expression values, defined as CT values between 38 and 40 cycles. All values below are rated as negative values (nd = not detectable). The *y-axis *represents expression levels normalized vs. rRNA. On the x-axis the chemokine receptors are indicated. CCR1, CCR2, CCR5, CCR7, CXCR4, CXCR5 and CX3CR1 are expressed in all of the tested samples. CCR4 is expressed in 21/22 tumors. CXCR3 is only detectable in a fraction of the tested samples and CCR10 is either borderline or not detectable. The overall receptor expression is higher in the tumor than in the bone precursors. The benign bone tumor (osteoblastoma) demonstrates expression values that are close to those of the sarcoma tissue.

The expression of specific chemokine receptors in the osteosarcoma tumor samples was first normalized to rRNA expression levels (see Figure 1) and then compared to the expression levels seen in normal bone samples, undifferentiated and differentiated fetal osteoblasts, primary mesenchymal stem cells, and an example of benign bone tumor.

CCR1, CCR2, CCR5, CCR7, CXCR4, CXCR5 and CX3CR1 were found to be expressed in 100% of the osteosarcoma samples tested. CCR4 was expressed in 21 of 22 samples. Low but detectable levels of CXCR3 mRNA were detected in 6 of 8 samples analyzed. Contrary to published reports, CCR10 mRNA expression was either not detected (signal was not detected by 40 cycles) (11/12) or was at the border of delectability (signal detected between cycles 38 and 40) (1/12). Importantly, control MSC and osteoblasts as well as the osteoblastic tumor sample showed detectable, and in case of MSC, functional expression of this receptor [15] (Figure 1). The osteoblastoma sample also showed expression of CCR1, CCR2, CCR4, CCR5, CCR7, CXCR4, CXCR5 and CX3CR1 at levels similar to those seen in the osteosarcoma biopsies. Chemokine receptor expression in mature cortical bone was generally only slightly above the detection limit (data not shown).

### Immunohistochemistry was used to verify the distribution of chemokine receptor expression in osteosarcoma samples

**Figure 2:**
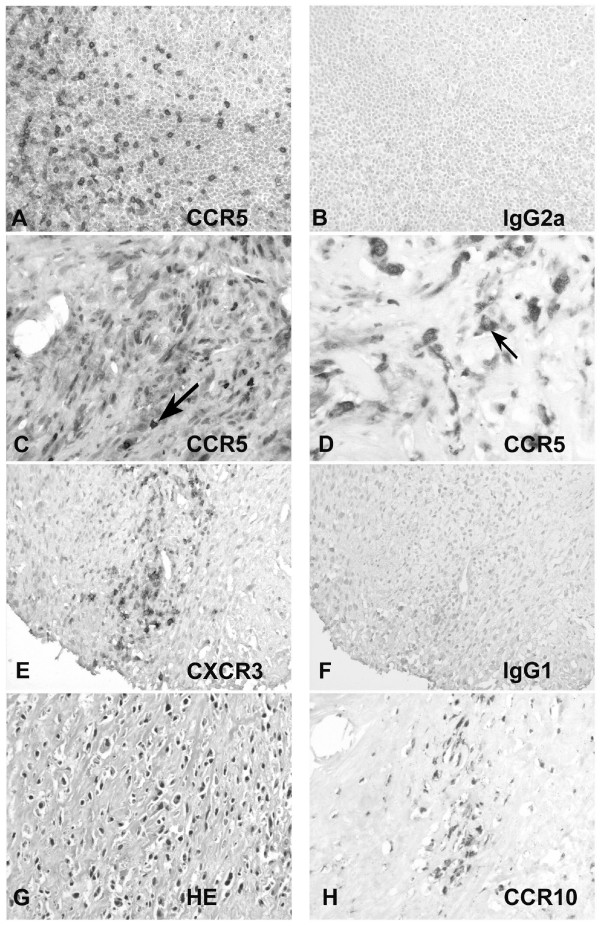
**Chemokine receptor expression pattern in the cellular context of osteosarcoma**. Antigen was retrieved from archival fixed sections of osteosarcoma samples. The samples were stained with monoclonal antibodies directed against CD3 (Becton Dickinson), CCR1, CCR4, CCR5, CCR7, CCR10 and CXCR3. (A) Immunostaining on human tonsil with a monoclonal AB directed against CCR5 (×200). (B) IgG2a isotype control for α-CCR5 on human tonsil. (C) Circumscriptive expression of CCR5 in a small follicular-like infiltrate (×400). (D) CCR5 staining of sections from human osteosarcoma (×400). Arrows label CCR5 positive infiltrating cells in (C) and CCR5 a positive tumor cell in (D) respectively. (E) Scattered expression of CXCR3 in very few mononuclear infiltrating cells (×400). (F) IgG1 isotype control for α-CXCR3 (×200). (G) HE-staining of a pleomorphic osteosarcoma with abundant osteoid production and highly pleomorphic, atypical mononuclear cells (×00). (H) Rare single infiltrating cells stain positive for CCR10 (×200).

**Figure 3:**
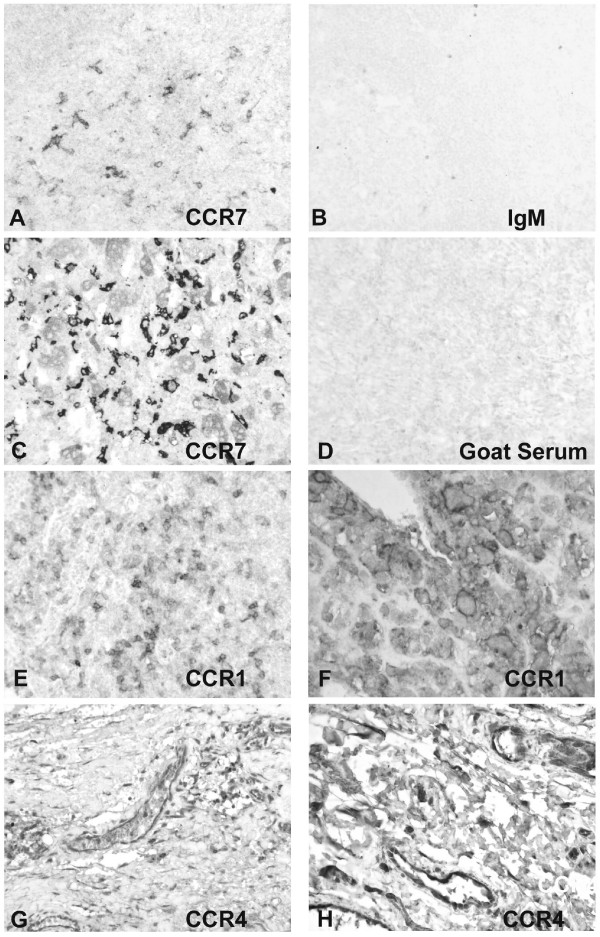
**Chemokine receptor expression pattern in the cellular context of osteosarcoma**. (A) Immunostaining on human tonsil with a monoclonal AB directed against CCR7 (×200). (B) IgG2a isotype control for α-CCR7 on human tonsil. (C) Expression of CCR7 in atypical mononuclear cells and some giant cells between some filigree and arborizing osteoid (×400). (D) Goat serum isotype controle for α-CCR4 and α-CCR1 on osteosarcoma sections. (E) and (F) Polynuclear giant cells express CCR1 as well as atypical mononuclear cells (E) ×200 and F) ×400). (G) and H) Strong CCR4 expression on cells of the inner lining of intratumoral vessels ((G) ×200 and (H) ×400).

Chemokine biology is thought to have both positive and negative effects on tumor growth depending on the biologic context of expression. To better characterize the distribution of chemokine receptors in osteosarcoma, immunohistochemistry using receptor specific antibody reagents was applied. Chemokine receptor expression was verified on nine representative archived osteosarcoma biopsies. The receptors analyzed included CCR1, CCR4, CCR5, CCR7, CCR10, CXCR3, and CXCR5. Antibodies for the additional receptors were either not available or could not be used for immunohistochemistry on paraffin embedded samples. Figure 2 and Fig 3 show representative osteosarcoma biopsy samples displaying the staining patterns seen for the various chemokine receptors. HE staining of the tumor (Figure 2 (G)) shows the histology of an osteoblastic osteosarcoma with extensive osteoid production and polymorphic tumor cells. The sections were stained in parallel for CD3 to distinguish between infiltrating T cells and tumor cells. All tumor samples tested showed a scattered infiltration by CD3 positive T-cells cells (data not shown). Expression of CCR1 was found in all tumor samples predominantly on polynucleated giant cells of benign origin, but also on a small number of tumor cells (Fig 3E and 3F). Antibody directed against CCR4 reproducibly stained the inner lining of small intratumoral vessels (Fig 3 (G) and 3 (H)). CCR5 was strongly expressed in all analyzed osteosarcoma biopsies on tumor cells (Fig 2 (D)) as well as infiltrating T-cells (Fig 2 (C)). CCR7 expression was demonstrated primarily on the infiltrating inflammatory cells of each analyzed sample and in only rare instances on a small subset of small dismorphic tumor cells (Fig 3 (C)). CXCR3 was detected on few scattered infiltrating mononuclear cells, but not on tumor cells, (Fig 2 (E)). The antibody directed against CCR10 detected only very rare positive infiltrating cells in one tumor sample (Fig 2 (H)) consistent with the mRNA data. CXCR5 protein expression could not be shown in any of the samples (data not shown) even though CXCR5 mRNA expression could be detected in the samples (Figure 1). Comparative statistics with regards to outcome could not be performed, because detectable expression of most receptors was found in all samples.

## Discussion

Osteosarcoma is characterized by a number of complex cytogenetic abnormalities and biological features, which have generally not yet been shown to be clinically relevant as diagnostic or prognostic markers. Further characterization of osteosarcoma is needed to aid in therapeutic strategies [2,23]. Chemokine receptors and their ligands have been implicated in the progression and metastasis of various tumors [5,7]. CCR7 mRNA expression has recently been correlated with metastasis free survival in osteosarcoma patients. CCR7 is known to be expressed by immature or central memory T cells, B cells and mature dendritic cells [4]. Interestingly, our data show that CCR7 is expressed primarily on the mononuclear infiltrate associated with the tumor and is only very rarely expressed by the tumor cells. This observation may help explain why osteosarcoma rarely metastasizes to lymph nodes as CCR7 is associated with leukocyte as well as metastatic tumor homing to secondary lymphatic organs and supports its association with the metastasis free survival previously described [24].

CXCR4 expression has been associated with the aggressiveness of osteosarcoma [25]. Unfortunately, CXCR4 specific antibody reagents that worked reproducibly on archival samples were not available and thus the distribution of expression could not be established. A recent report by Oda et al. examining CXCR4 expression in frozen osteosarcoma tumor samples suggests that CXCR4 is found to be higher expressed on the tumor cells in the metastatic site as compared to the primary tumor [25]. It is important to remember that the CXCR4 receptor is expressed by virtually all leukocytes thus the level of mRNA expression detected in the primary or metastatic tumor will be due at least in part to the extent of mononuclear infiltration present [4].

Both tumor cells and mononuclear infiltrates were found to strongly express CCR5. The level of CCR5 expression by tumor cells did however differ between individual samples. CCR5 expression has been implicated in tumor progression [11] and has been linked to angiogenesis [9] but has not been previously described in osteosarcoma. Microvessel density in the tumor, representing the level of neoangiogenesis, has been associated with clinical outcome in osteosarcoma [26]. The small number of samples did however not allow the analysis of the correlation between microvessel density and CCR5 expression. This association could be of potential therapeutic interest since CCR5 Antagonists are currently under phase III clinical evaluation (4).

CCR4 and its ligands (CCL3, CCL17 and CCL22) are thought to contribute to the formation of vascular structures by mobilizing smooth muscle precursor cells [27]. CCR4 receptor expression in our samples was localized to the inner lining of intratumoral vessels by immunohistochemistry. CCR4 has also been previously described on vascular endothelial cells in brain and heart [28]. Although tumor vessels normally do not show a distinct smooth muscle layer, CCR4 expression in the osteosarcoma samples is suggestive of an involvement of this receptor in neoangiogenesis in this tumor.

The recruitment of leukocytes in acute and chronic inflammatory processes is in part regulated by CCR1 [4]. While diverse mononuclear cells express CCR1, its expression has also been described on tumor cells [4,8,29,30]. Here intratumoral polynuclear giant cells predominantly expressed CCR1 protein. These cells are frequently found in osteosarcoma and probably represent osteoclasts derived from macrophages [2]. Their function in the tumor context is at present unknown. They might contribute to the recruitment of mononuclear infiltrating cells into the tumor and thus the anti-tumor response. A potential link of the number of giant cells and prognosis could be analyzed given a larger number of samples.

The chemokine receptor CXCR3 is expressed by activated T cells, NK cells and a subpopulation of monocytes (4). In the osteosarcoma samples tested, CXCR3 was found expressed on scattered mononuclear infiltrating cells. The low number of expressing cells was also reflected in the low level of mRNA expression detected by TaqMan analysis.

Expression of the CCR10 receptor in osteosarcoma has been previously associated with clinical parameters [12]. In the study performed here, no significant CCR10 expression was found at either the mRNA or protein level except for rare infiltrating mononuclear cells in one patient sample. However, CCR10 mRNA and protein was easily detected in the bone derived control cells suggesting that the reagents were adequate for detection of this receptor [18]. These results suggest that previously published reports of an association of CCR10 mRNA expression with clinical parameters in osteosarcoma patients may have to be reinterpreted, and emphasizes the importance of verifying mRNA expression using immunohistochemistry when possible.

The level of chemokine receptor expression was generally found to be several orders of magnitude higher in the tumor samples as compared to the levels seen in the control differentiated and undifferentiated osteoblasts or MSC. However, the observation that a benign bone tumor expressed chemokine receptor mRNA at approximately the same level as malignant tumors suggests that chemokine biology may not be crucial for the transformation of the bone precursors, but may still play a role in tumor progression via contribution to neoangiogenesis as well as in the propagation or spreading of tumor cells. Care should be taken in the interpretation of the results presented as differences in the fixation process of human samples or control tissues could in part influence the results of the real time PCR analysis. However, the general trend of chemokine receptor expression is clear. A larger cohort of osteosarcoma patient samples should allow for more specific conclusions and the inclusion of potential prognostic variables.

## Conclusion

The results detailed here refine our understanding of the expression of chemokine receptors in osteosarcoma. Clearly chemokine receptors may play diverse roles in the pathology of this tumor. The significance of each receptor with regards to the course and outcome of the disease should be evaluated in a larger cohort of patient samples. Our results emphasize the importance of identifying the cellular source of the detected chemokine receptor. This is clearly a central issue when interpreting the significance of specific findings for the application of prognostic markers or the characterization of therapeutic targets.

## Competing interests

The author(s) declare that they have no competing interests.

## Authors' contributions

IvL designed the study, analyzed the data, interpreted the data and drafted as well as revised the manuscript. SS carried out the immunohistology on the tumor samples and revised the manuscript. AW carried out the RT-PCR and helped analyzing the data. MNoto contributed in acquisition of the data and analysis. MN contributed the tumor samples and clinical data as well as revised the manuscript. MK carried out the microdissection of the tumor samples and histopathological analysis. AH contributed significantly in analyzing and interpreting the PCR data and revised the manuscript. RD was involved in critical revision of the manuscript and the experimental design. SB revised the manuscript. RH helped design the study, carried out the pathohistological analysis of the tumor samples and revised the manuscript. PJN contributed to design, analysis and interpretation of the data and revised the manuscript critically for important intellectual content. All authors have read and approved the final version of the manuscript.

## Pre-publication history

The pre-publication history for this paper can be accessed here:


